# Speciation of Arsenic in Exfoliated Urinary Bladder Epithelial Cells from Individuals Exposed to Arsenic in Drinking Water

**DOI:** 10.1289/ehp.11503

**Published:** 2008-07-18

**Authors:** Araceli Hernández-Zavala, Olga L. Valenzuela, Tomás̆ Matous̆ek, Zuzana Drobná, Jir̆í Dĕdina, Gonzalo G. García-Vargas, David J. Thomas, Luz M. Del Razo, Miroslav Stýblo

**Affiliations:** 1 Center for Environmental Medicine, Asthma, and Lung Biology, University of North Carolina at Chapel Hill, Chapel Hill, North Carolina, USA; 2 Sección de Toxicología, Centro de Investigación y de Estudios Avanzados del Instituto Politécnico Nacional, México DF, México; 3 Institute of Analytical Chemistry of the ASCR, v.v.i., Prague, Czech Republic; 4 Department of Nutrition, University of North Carolina at Chapel Hill, Chapel Hill, North Carolina, USA; 5 Facultad de Medicina, Universidad Juárez del Estado de Durango, Gómez Palacio, Durango, México; 6 Pharmacokinetics Branch, Experimental Toxicology Division, National Health and Environmental Effects Research Laboratory, Office of Research and Development, U.S. Environmental Protection Agency, Research Triangle Park, North Carolina, USA

**Keywords:** arsenic species, drinking water, exfoliated human urinary bladder epithelial cells

## Abstract

**Background:**

The concentration of arsenic in urine has been used as a marker of exposure to inorganic As (iAs). Relative proportions of urinary metabolites of iAs have been identified as potential biomarkers of susceptibility to iAs toxicity. However, the adverse effects of iAs exposure are ultimately determined by the concentrations of iAs metabolites in target tissues.

**Objective:**

In this study we examined the feasibility of analyzing As species in cells that originate in the urinary bladder, a target organ for As-induced cancer in humans.

**Methods:**

Exfoliated bladder epithelial cells (BECs) were collected from urine of 21 residents of Zimapan, Mexico, who were exposed to iAs in drinking water. We determined concentrations of iAs, methyl-As (MAs), and dimethyl-As (DMAs) in urine using conventional hydride generation-cryotrapping-atomic absorption spectrometry (HG-CT-AAS). We used an optimized HG-CT-AAS technique with detection limits of 12–17 pg As for analysis of As species in BECs.

**Results:**

All urine samples and 20 of 21 BEC samples contained detectable concentrations of iAs, MAs, and DMAs. Sums of concentrations of these As species in BECs ranged from 0.18 to 11.4 ng As/mg protein and in urine from 4.8 to 1,947 ng As/mL. We found no correlations between the concentrations or ratios of As species in BECs and in urine.

**Conclusion:**

These results suggest that urinary levels of iAs metabolites do not necessarily reflect levels of these metabolites in the bladder epithelium. Thus, analysis of As species in BECs may provide a more effective tool for risk assessment of bladder cancer and other urothelial diseases associated with exposures to iAs.

Arsenic, a naturally occurring toxic metalloid, is widely distributed in the environment. Inorganic As (iAs), a chemical form of As commonly found in drinking water supplies (Irgolic 1994), is one of the most potent environmental carcinogens. Worldwide, millions of people suffer from cancer and other diseases associated with consumption of water containing high levels of iAs [Agency for Toxic Substances and Disease Registry (ATSDR) 2007]. The conditions produced by chronic poisoning with iAs are generally referred to as arsenicosis. Population studies in arsenicosis-endemic areas have consistently shown that susceptibility to chronic toxicity of iAs varies even among individuals at similar exposure levels. Evidence from population, clinical, and laboratory studies suggests that this variation is due, at least in part, to interindividual differences in iAs metabolism.

In humans, iAs is metabolized to methyl-As (MAs) and dimethyl-As (DMAs) species that contain either As^III^ or As^V^ (Cullen et al. 1984; Devesa et al. 2004). Arsenic (+3 oxidation state) methyltransferase (AS3MT) (Lin et al. 2002) is the key enzyme in this metabolic pathway (Drobná et al. 2006; Thomas et al. 2007). To date, four methylated arsenicals have been identified as major metabolites of iAs in humans: methylarsonous acid (MAs^III^), methylarsonic acid (MAs^V^), dimethylarsinous acid (DMAs^III^), and dimethylarsinic acid (DMAs^V^). These methylated arsenicals and the iAs species arsenate (iAs^V^) and arsenite (iAs^III^) are consistently detected in urine of individuals exposed to iAs in drinking water (Aposhian et al. 2000; Del Razo et al. 2001; Le at al. 2000; Mandal et al. 2001; Valenzuela et al. 2005). In addition, a sulfur-containing derivative of DMAs^V^, dimethylthioarsinic acid (DMTA), has recently been found in urine of residents in arsenicosis-endemic areas of Bangladesh (Raml et al. 2007). The concentrations and ratios of these As species in urine vary significantly among individuals (Vahter 1999; Yu et al. 2000). We and others have shown that MAs^III^ or DMAs^III^ are more potent than iAs species or MAs^V^ and DMAs^V^ as cytotoxins, genotoxins, enzyme inhibitors, and modulators of major signal-transduction pathways (Drobná et al. 2003; Mass et al. 2001; Paul et al. 2007; Petrick et al. 2001; Styblo et al. 2002; Thomas et al. 2001). Interrelations in the metabolism of oxo- and thioarsenicals have not been resolved. However, DMTA is more toxic to mammalian cells than the pentavalent methylated arsenicals (Naranmandura et al. 2007). Thus, differences in the formation, tissue retention, and clearance of As^V^- and As^III^-containing metabolites of iAs are likely to contribute to interindividual variations in susceptibility to adverse effects associated with iAs exposures.

Previous studies have shown that ratios of As species (e.g., MAs/iAs or DMAs/MAs) in urine are associated with prevalence of skin lesions among residents of arsenicosis-endemic areas of Taiwan and Mexico (Chen et al. 2003; Del Razo et al. 1997; Valenzuela et al. 2005; Yu et al. 2000). Similar associations have been reported between urinary levels of iAs metabolites and the concentration of transforming growth factor-α (TGF-α), a potential marker of urinary bladder cancer, in exfoliated bladder epithelial cells (BECs) isolated from urine of individuals chronically exposed to iAs in drinking water (Valenzuela et al. 2007). However, the utility of urinary As species for assessment of health risks associated with the exposure to iAs remains uncertain. The magnitude and character of adverse effects of iAs exposure are ultimately determined by the concentration and chemical form of As in target tissues. Therefore, analysis of total or speciated As in these tissues could provide an effective tool for estimating internal exposure and for identification of individuals with an increased risk of developing specific diseases associated with chronic exposures to iAs.

Development of sensitive techniques for analysis of trivalent and pentavalent As species in complex biological matrices, including human cells and tissues, is a prime requirement for better understanding of dose–response relations for iAs in humans. We have recently optimized instrumentation and methods for speciation analysis of As by hydride generation-atomic absorption spectrometry using a cryotrap (HG-CT-AAS) for capture and separation of arsines, and a multiple microflame quartz tube atomizer (multiatomizer) for a more efficient atomization of As (Matous ek et al. 2002, 2008). We used the optimized HG-CT-AAS in the present study for analysis of As species in BECs isolated from individuals chronically exposed to iAs in drinking water. Our results show that both urine and BECs contain iAs and its methylated metabolites MAs and DMAs. However, we found no correlations between the concentrations or ratios of As species in urine and BECs. These findings suggest that speciation of As in BECs rather than in urine should be used for risk assessment of diseases affecting urinary bladder or urothelium of individuals chronically exposed to iAs.

## Materials and Methods

### Study subjects

Twenty-one individuals (19 females and 2 males; 14–64 years of age) were randomly selected from 196 subjects involved in an ongoing population study in Zimapan, Mexico. Concentrations of iAs in drinking water from wells used by these subjects ranged from < 1 to 190 μg As/L. The protocol for this study was approved by the Human Research Ethics Boards of the University of North Carolina (UNC) at Chapel Hill and of Centro de Investigación y de Estudios Avanzados del Instituto Politécnico Nacional (CINVESTAV), Mexico. All participants gave written informed consent.

### Collection of urine and isolation of BECs

Spot urine samples were collected in acid-washed polypropylene containers in local health department facilities in Zimapan. The containers were packed in ice and transported to CIN-VESTAV laboratories, which are located in Mexico City, about a 2-hr drive from Zimapan. Here, each urine sample was divided into two or three 50-mL polyethylene tubes. BECs were isolated by centrifugation at 300 × *g* for 10 min at 4°C. Cells from each donor were then transferred into a single conical 1.5-mL Eppendorf tube, washed with ice-cold phosphate-buffered saline (PBS), and centrifuged at 300 ×*g* for 5 min at 4°C. Cells were washed again with PBS and pelleted by centrifugation. The pellets were packed in dry ice and air-shipped to UNC-Chapel Hill. Here, the pellets were stored for several days at −80°C before analysis. Aliquots of urine were stored at −75°C until analyzed at CINVESTAV.

### Analysis of As species in urine and BECs

We analyzed arsenic species in urine by HG-CT-AAS using a PerkinElmer Model 3100 AA spectrometer (PerkinElmer, Norwalk, CT, USA) equipped with a conventional quartz tube atomizer (Del Razo et al. 2001). Hydrides (i.e., arsine and the methyl-substituted arsines) were generated in a reaction with sodium borohydride (NaBH_4_; EM Science, Gibbstown, NJ, USA) in the presence of concentrated HCl (Sigma-Aldrich, St. Louis, MO, USA). Under these conditions, hydrides are generated from both trivalent and pentavalent As species (Del Razo et al. 2001; Devesa et al. 2004). We analyzed As species in BECs by a recently developed automated HG-CT-AAS technique using a PerkinElmer Model 5100 PC AA spectrometer equipped with the multiatomizer and a FIAS200 flow injection accessory (Hernández-Zavala et al. 2008; Matous ek et al. 2008). Unlike the conventional HG-AAS used for the urine analyses, the new method provides low detection limits (DLs) needed for analysis of As species in small samples of BECs. Before analysis, each BEC pellet was lysed in 1.25 mL 0.5% solution of Triton X-100 (Sigma-Aldrich) in deionized water. BEC lysates were treated with 2% l-cysteine hydrochloride (EMD Chemicals Inc., Darmstadt, Germany) for 70 min at room temperature. Treatment with cysteine reduces all pentavalent As species to trivalency. Hydrides were generated from 0.5-mL aliquots of cysteine-treated samples by reaction with NaBH_4_ in a Tris-HCl (Sigma-Aldrich) buffer (pH 6) as previously described (Hernández-Zavala et al. 2008; Matous ek et al. 2008). HG-CT-AAS was developed for the oxidation-state–specific speciation analysis of As, but under current operating conditions both procedures described above determined total iAs (iAs^III^ + iAs^V^), MAs (MAs^III^ + MAs^V^), and DMAs (DMAs^III^ + DMAs^V^).

### Calibration and method validation

We used the following standards to generate calibration curves for quantification of iAs, MAs, and DMAs: iAs^V^, sodium salt, (96% pure; Sigma-Aldrich), MAs^V^, disodium salt (98% pure; Chem Service, West Chester, PA, USA), and DMAs^V^ (98% pure; Strem Chemicals, Inc., Newburyport, MA, USA). Standard solutions for quantification of As species in urine were prepared in deionized water. For quantification of As species in BECs, the standards solutions were spiked into Triton X-100 lysates of human hepatocellular carcinoma (HepG2) cells (American Type Culture Collection, Manassas, VA, USA). Identities of arsines generated from urine and BECs were confirmed by spiking samples with As standards at several concentrations.

Concentrations of iAs, MAs, and DMAs were expressed as nanograms As per milliliter for urine and nanograms As per milligram protein for BECs. The protein concentrations in BEC lysates were determined using an RC DC Protein Assay kit (BioRad, Hercules, CA, USA); bovine serum albumin was used for assay calibration. We used standard reference material (SRM) 2670a urine (National Institute of Standards and Technology, Gaithersburg, MD, USA), with a reference value for total As concentration of 220 μg/L, for validation of urine analyses. The sum of As species (mean ± SD; *n* = 3) determined in SRM 2670a urine by conventional HG-CT-AAS (207 ± 6 μg As) was in good agreement with the reference value for total As content. There are no SRMs for analysis of total As or As species in cells or tissues. However, our previous studies showed that the recoveries of As species from human cell lysates pretreated with cysteine and analyzed by the automated HG-CT-AAS with the multiatomizer range from 88 to 98% (Hernández-Zavala et al. 2008).

### Statistical analyses

We used Stata 8.0 (Stata Corp., College Station, TX, USA) and Instat (GraphPad Software Inc., San Diego, CA, USA) statistical software packages to analyze results of this study. Differences in the percentages of As species and the species ratios between urine and BECs were evaluated by unpaired *t*-test. The nonparametric Spearman correlation was used to analyze associations between the concentrations of As species, the percentage of As species, or ratios of As species in urine and BECs. Differences or correlation with *p*-values < 0.05 were considered statistically significant.

## Results and Discussion

BECs exfoliated into urine originate in epithelium of the genitourinary tract including the urinary bladder. The basic characteristics of blood and urine (e.g., chemical composition, pH, osmolality) differ significantly. The apical surface of the bladder epithelium represents an effective permeability barrier that maintains large chemical and electrical gradients between the urine and the blood (Hicks et al. 1974). In humans, turnover of superficial BECs takes about 200 days (Born et al. 2003). Thus, BECs isolated by catheterization and washing of the bladder epithelium or BECs collected from voided urine are a unique material for study of metabolic or genetic changes in human urothelium caused by diseases or by long-term exposures to toxins or carcinogens. Notably, BECs exfoliated to the urine are viable and can be cultured (Dörrenhaus et al. 2000; Herz et al. 1993). Diagnostic tests on exfoliated BECs (e.g., analyses of tumor antigens, nuclear matrix proteins, fibrin degradation products, telomerase activity) have the potential to replace cystoscopies or resections in the diagnosis of bladder tumors (Ozen 1999). BECs have also been used to measure cytogenetic end points in studies evaluating genotoxic effects of chemical carcinogens targeting urinary bladder, including iAs (Murray and Edwards 1999; Warner et al. 1994). An exposure-dependent increase in micro-nucleated BECs was reported among individuals exposed to iAs in drinking water (Biggs et al. 1997; Moore et al. 1997). However, because of limitations of methods used for analysis of As species in biological samples, association between the micronucleus formation or other pathologic processes and the concentrations of iAs metabolites in BECs has not been examined.

**Figure 1:**
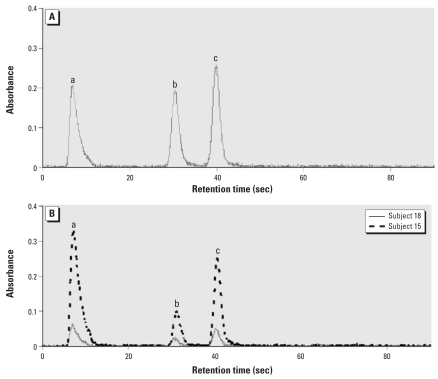
HG-CT-AAS analysis of As standards and As species in BECs showing separation and detection of arsine (a), methylarsine (b), and dimethylarsine (c) generated from a mixture of As standards, iAs^V^, MAs^V^, and DMAs^V^ (0.5 ng As each), spiked into a HepG2 cell lysate (*A*) and from BECs isolated from two subjects included in this study (*B*). The mixture of standards and BEC lysates were treated with cysteine to allow arsine generation from both trivalent and pentavalent As species.

HG-CT-AAS is uniquely suited for quantitative analysis of As species in complex biological matrices in which arsenicals are bound to protein or low-molecular-weight thiols. Some of these matrices, specifically cell lysates, can be analyzed directly without prior digestion or extraction, thus limiting oxidation of the unstable methylated As^III^-containing species. We have recently optimized this technique, increasing its sensitivity considerably for all relevant As species and improving its throughput (Hernández-Zavala et al. 2008; Matous ek et al. 2008). The optimized HG-CT-AAS was used in the present study for speciation analysis of As in BECs collected from individuals chronically exposed to iAs in drinking water. To match the matrix of BEC cells, As standards used for calibration were prepared in HepG2 lysates at final concentrations of 0.1–5 ng As/mL. The calibration curves for iAs^V^, MAs^V^, and DMAs^V^ standards were linear over the entire concentration range (Table 1); the DL values were 12 pg As for iAs^V^, 17 pg As for MAs^V^, and 13 pg As for DMAs^V^. Precision of the method expressed as relative SD for three As standard concentrations (0.25, 0.5, and 1 ng) ranged from 0.7 to 3.1% (data not shown). Figure 1 shows examples of AAS signals for arsine, methylarsine, and dimethylarsine generated from a mixture of As standards (iAs^V^, MAs^V^, and DMAs^V^) prepared in HepG2 lysates and from BEC samples (donors 15 and 18) pre-treated with cysteine. In both cases, complete baseline separations of arsine, methylarsine, and dimethylarsine were achieved. The average retention times (mean ± SD) were 7.8 ± 0.11 sec for arsine, 30 ± 0.38 sec for methylarsine, and 39.3 ± 0.4 sec for dimethylarsine.

**Figure 2:**
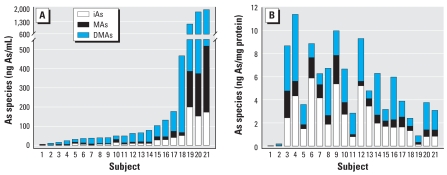
Distribution of As species in urine (*A*) and BECs (*B*) from 21 study subjects exposed to iAs in drinking water. Urines and BEC lysates were treated with cysteine to allow arsine generation from both trivalent and pentavalent As species. Levels of As species in BECs from subject 1 were < DLs of the method.

Table 2 and Figure 2 summarize the speciation analysis of As in BECs and urine. iAs, MAs, and DMAs were present in all 21 urine samples. The sum of As species in urine ranged from 4.8 to 1,947 ng As/mL. DMAs was the major As species, accounting for an average of 69% of iAs metabolites in urine. iAs, MAs, and DMAs were also detected in 20 of 21 BEC samples. The levels of As species in BECs from donor 1 were < DLs of the method. The sum of As species in BECs ranged from 0.18 to 11.4 ng As/mg protein. On average, iAs represented a significantly greater fraction of As species in BECs (42%) compared with urine (17%). On average, DMAs accounted for about 43% of As species in BECs. MAs was a minor metabolite in both BECs and urine, accounting for an average of 15% and 13%, respectively. Consequently, the MAs/iAs, DMAs/MAs, and DMAs/iAs ratios were significantly greater in urine than in BECs. The ratios of As metabolites in human urine have frequently been interpreted as indicators of the efficiency of iAs methylation or as markers of the individual capacity to methylate iAs. However, the actual associations between the As metabolites excreted in urine and those retained in tissues have never been properly examined. In this study, no statistically significant correlations were found between the sum of As species in urine and BECs or between the concentrations of the individual As species or the ratios of As species in urine and BECs. Similarly, no statistically significant correlations were found between the age of study subjects and the concentrations, percentages, or ratios of As species in urine or BECs.

Our results show that the levels and composition of iAs metabolites in BECs do not reflect those found in urine. Several factors could affect the concentrations and speciation of As in BECs after separation from bladder epithelium, including *a*) the integrity of the plasma membrane, *b*) the activity of cellular transport systems regulating the influx and efflux of As species in BECs, *c*) the capacity of BECs to methylate iAs, and *d*) the binding of As species to high-affinity targets in BECs. Previous work showed that BECs collected at the same study site in Zimapan and processed using the same protocol maintained morphologic integrity (Valenzuela et al. 2007). This observation is consistent with results of clinical studies (Dörrenhaus et al. 2000; Herz et al. 1993) in which BECs were used to examine potential biomarkers of bladder cancer. We have previously shown that SV-40–transformed normal human urinary bladder epithelial (UROtsa) cells do not express AS3MT and do not methylate iAs (Drobná et al. 2005). AS3MT expression and activity in BECs have not yet been examined. Cultured UROtsa cells exposed to equimolar concentrations of iAs^III^, MAs^III^, or DMAs^III^ retained MAs^III^ and DMAs^III^ more avidly than iAs^III^ (Drobná et al. 2005), indicating that uptake or binding processes in these cells favor methylated trivalent arsenicals. However, in the present study, iAs represented a major fraction of As species retained in BECs but was only a minor As species in urine. These data suggest that the affinity of BECs for iAs and methylated As species may differ from that of cultured UROtsa cells. The multidrug resistance-associated protein-1 and -2 and P-glycoprotein, which mediate transport of arsenicals in cell culture systems (Kala et al. 2000; Liu et al. 2001, 2006; Steele et al. 2005), are expressed in UROtsa cells (Drobná et al., unpublished data). Future studies will examine the expression and activities of these transporters in BECs and the roles of these transporters in accumulation of specific As species in these cells.

## Conclusions

The results of this study confirm that the optimized HG-CT-AAS is suitable for speciation analysis of As in small samples of exfoliated BECs. Notably, these results also suggest that the concentration and speciation of As in BECs do not correlate with the concentration and speciation of As in urine. The urinary profiles of As species have been shown to reflect only a recent exposure to iAs; however, the concentrations of As species in BECs are likely to reflect the integrated exposure over a period of about 200 days, the lifetime of the superficial layer of bladder epithelium (Born et al. 2003). Thus, analysis of As species in BECs may provide a more effective tool for risk assessment of bladder cancer and, possibly, other diseases associated with chronic exposure to iAs. The optimized HG-CT-AAS will be used in our future studies for the oxidation state specific analysis of As species in BECs with focus on the most toxic metabolites of iAs: MAs^III^ and DMAs^III^. In addition, we plan to examine association between the concentrations of As species in BECs and markers of the adverse effects of iAs exposure, including micronucleus formation and TGF-α concentration in BECs and skin lesions.

## Figures and Tables

**Table 1:** The characteristics of the calibration curves and DLs for As species analyzed by the optimized HG-CT-AAS.

As standards	Linear regression^a^	Correlation coefficient^a^	DL (pg As)^b^
iAs^V^	*y* = 1.04*x* + 0.041	0.999	12
MAs^V^	*y* = 0.94*x* + 0.02	0.999	17
DMAs^V^	*y* = 1.03*x* + 0.046	0.999	13

a Calibration curves were generated for As standards spiked into a HepG2 cell lysate at final concentrations of 0.1–5 ng As/mL; all standards were treated with cysteine before HG-CT-AAS analysis.

b DLs were calculated as 3 sigma for the area of blanks (*n* = 10) at retention times corresponding to each of the As species.

**Table 2:** Arsenic species in exfoliated BECs (ng As/mg protein) and urine (ng As/mL).

	BECs		Urine	
	Mean ± SD	Range	Mean ± SD	Range
iAs	2.41 ± 1.75	0.06–5.89	36.6 ± 60.2	1–201
MAs	0.80 ± 0.66	0.03–2.43	42.3 ± 90.2	1–342
DMAs	2.19 ± 1.5	0.10–5.75	221.5 ± 440.5	2.8–1,449
iAs+MAs+DMAs	5.41 ± 3	0.18–11.35	300.5 ± 583.5	4.8–1,947
%iAs	42 ± 16.1*	20–72.8	17 ± 7.7	8.4–35.0
%MAs	15 ± 5.6	4.1–26.3	13 ± 6.1	2.6–30.1
%DMAs	43 ± 17.6*	13.7–65	69 ± 12.3	36.8–85.9
MAs/iAs	0.4 ± 0.21*	0.07–0.92	0.8 ± 0.40	0.1–2.0
DMAs/MAs	3.8 ± 2.75*	0.70–10.5	7.8 ± 7.4	1.2–29.6
DMAs/iAs	1.3 ± 0.90*	0.19–3.2	5.0 ± 2.6	1.1–9.5

Results are shown for 21 urine samples and 20 BEC samples; levels of As species in one BEC sample were < DLs of the method.

* Value in BECs is significantly different (*p* < 0.05) compared with the corresponding value in urine.
